# Longitudinal Analyses of Mental Health in Autistic Individuals: A Systematic Review

**DOI:** 10.3390/brainsci14101033

**Published:** 2024-10-18

**Authors:** Maira Tafolla, Catherine Lord

**Affiliations:** Semel Institute for Neuroscience and Human Behavior, University of California Los Angeles, 760 Westwood Plaza, Los Angeles, CA 90024, USA; mtafolla@mednet.ucla.edu

**Keywords:** autism, anxiety, depression, longitudinal, trajectory

## Abstract

Background/Objectives: Co-occurring mental health conditions affect autistic individuals at high rates, impacting their well-being and quality of life. Mental health conditions are often appropriate treatment targets that can improve the lives of autistic individuals. Because of this, there is growing interest in predictors of mental health and behavioral outcomes. Given the rapidly evolving evidence base and growing literature using longitudinal cohorts, it is unclear which predictors of symptoms of anxiety and depression are consistent, and which are not. Additionally, it is difficult to deduce which predictors of mental health symptoms at a given time also predict change over time. This can be partially due to the different statistical approaches that are implemented, including trajectory vs. non-trajectory methodologies. Methods: We conducted a systematic review to evaluate how non-trajectory and trajectory analyses inform our knowledge of how symptoms of anxiety and depression change over time. Additionally, we aimed to identify important predictors of change and later anxiety and depressive symptoms in autistic individuals. Results: There is variability in symptoms of anxiety and depression in autistic individuals. Adaptive skills arose as significant predictors of change and of later symptoms of both anxiety and depression. Peer relationships in school age seem to be particularly important in predicting later symptoms of depression. Conclusions: This review provides evidence that there are different trajectories and different patterns of mental health symptoms over the lifespan, providing further evidence that autism is a developmental condition that changes over time in different ways for different people. Implications and future directions are further discussed.

## 1. Introduction

Autism is a neurodevelopmental condition diagnosed based on social communication difficulties and restricted and repetitive interests and behaviors that are present early in development and significantly impact functioning [1]. Autism often co-occurs with other neurodevelopmental (e.g., intellectual disability, attention-deficit hyperactivity disorder [ADHD]), medical, psychological, and behavioral (e.g., aggression) conditions. For instance, an estimated 26–37.9% of autistic individuals have a co-occurring intellectual disability [2,3]. Higher rates of co-occurring conditions affect autistic individuals compared to the general population [4] and can impact the mental health and well-being of autistic individuals throughout their lives [5]. This study focuses specifically on symptoms of anxiety and depression due to the high rates of their co-occurrence with autism and the significance to stakeholders. Mental health and psychological well-being have been defined in many ways in the literature. One conceptualization encompasses psychological/cognitive, emotional, and behavioral well-being.

### 1.1. Mental Health Disorders in Autism

Symptoms of depression and anxiety are particularly common in autistic individuals. However, identifying mental health disorders such as depression and anxiety in autistic individuals can be challenging. Many of the symptoms of autism and these psychological conditions overlap (also known as symptom over-shadowing) and can be difficult to disentangle from one another [6]. For example, challenges in social communication are common in both anxiety and autism [7]. Additionally, only a limited number of anxiety and depression measures have been validated for autistic people [6,8,9]. Consequently, estimates of mental health issues in autistic individuals vary based on diagnostic procedures, measures, and sampling differences [10]. While estimates of co-occurring mental health rates vary across the literature, the rates of mental health challenges that affect autistic people are still consistently higher compared to rates in neurotypical individuals [4]. Estimates from two studies suggested that current anxiety prevalence estimates are 7.8% in autistic children, 21.5% in autistic adolescents, and 27% in autistic adults [3,6]. When considering the prevalence rates of anxiety in autistic adults who have a co-occurring intellectual disability (ID), the pooled estimates are lower (20%) [6]. Depression prevalence rates in autism from the same studies are 1% in autistic children, 12.7% in autistic adolescents, 23% in autistic adults without ID, and 16% in autistic adults with ID [3,6]. 

Cross-sectional designs are useful and cost-efficient tools to help researchers identify prevalence rates of co-occurring psychiatric conditions and their concurrent associations with predictors. Longitudinal designs are necessary to understand the trajectories of mental health symptoms over the lifespan, however, and to identify critical early predictors of later outcomes. Mental health outcomes are particularly important because they are associated with the overall well-being of autistic adults [5,6,8,11]. Using a large and diverse community sample (*n* = 194) McCauley and colleagues [5] found that having high and stable anxiety levels over time predicted worse objectively defined adult outcomes that were developed using a framework from the World Health Organization (having at least one friend, living independently, having a job) for adults with IQ scores greater than 70. A similar association was seen between higher levels of depression and worse adult outcomes [5]. A separate study using a smaller clinical sample (*n* = 58) found a positive correlation between individuals’ mental health and poor social outcomes in adulthood [8]. These studies suggest that mental health is a significant predictor of traditional objective adult outcomes above and beyond autism symptoms. It is important to understand the mechanisms that contribute to these mental health outcomes of autistic people over their lifespan.

### 1.2. Longitudinal Cohort Studies

Long-term longitudinal studies in autism are particularly helpful because they are less likely to be influenced by trends and changes in diagnostic criteria over time. For example, prevalence rates of autism have changed dramatically over the last 40 years, a phenomenon most often attributed to better awareness, but also to broader interpretations of autism definitions [12]. Thus, cross-sectional comparisons of recently diagnosed young children with later diagnosed or early diagnosed adolescents or adults may be difficult to interpret. Though participants in longitudinal studies starting in early childhood likely represent a different population of individuals than current cross-sectional studies (with children diagnosed at younger ages likely to have substantial developmental delays), at the least the participants are compared to themselves as they grow older.

Moreover, different statistical methods can be implemented in longitudinal studies to understand data and trends over time in the symptoms measured. Trajectory methods can be particularly useful in elucidating change over time, since they are focused on patterns of change across multiple developmental periods. Additionally, trajectory methods assume the population is heterogeneous and subgroups follow different developmental trajectories. In contrast, non-trajectory methods assume the population is homogeneous, and unlike trajectory models, do not elicit different patterns of change. Non-trajectory methods used in longitudinal studies can provide information about the causal relationship between the average of an earlier construct and the average of a later outcome for the entire sample.

Trajectory methods help us understand how symptoms of anxiety and depression evolve over time, identifying critical periods in autistic individuals’ lives when support is especially important. For example, a study using an ongoing longitudinal sample (*n* = 165) collected anxiety and depressive symptom questionnaires from autistic individuals during several timepoints, including school age, adolescence, and early adulthood [13]. Results showed that males had higher symptoms of anxiety and depression than females during school age (entry timepoint), but females showed greater increases in symptoms of anxiety and depression over time throughout adolescence, which resulted in no significant differences in symptoms between males and females at age 21. These results provide important clinical guidance, suggesting that males with autism may experience internalizing symptoms earlier in development, while females show sharp increases in symptoms during adolescence. It may be worth screening for anxiety and depression earlier in development in order to equip all autistic individuals, but especially females, with the tools to offset symptoms of anxiety and depression prior to adolescence.

Identifying consistent predictors of long-term mental health outcomes in autistic adults is also important for several reasons. Longitudinal studies assessing predictors can help identify early clinical targets that can influence positive mental health outcomes, and overall well-being. For instance, a study using a large community sample of autism (*n* = 390) found a significant and positive association between caregiver depressive symptoms at age 3 and their autistic child’s depressive symptoms at age 14 [14]. In the same study, peer relationships at age 10 were identified as significant mediators between early childhood irritability and depression at age 14 in autistic individuals [14]. These findings can help to inform targeted interventions to best support youth. For example, intervening more directly on peer relationships at age 10 could protect against later mental health challenges affecting autistic adolescents, based on these study findings. Other early predictors of later mental health outcomes in autism include sociodemographic and contextual factors (i.e., gender and caregiver education [used as a proxy for SES]), peer-relationships, parental well-being, and parent–child interaction styles [13,14,15,16]).

### 1.3. Aims

The increasing number of large longitudinal cohorts and growing interest in predictors of mental health and behavioral outcomes is encouraging, given their importance to stakeholders, including autistic individuals and their families [17]. Given the rapidly evolving evidence base, it is not clear which predictors of anxiety and depression are most important, and which are not. Additionally, it is difficult to deduce which predictors of mental health symptoms at a given time also predict change over time. This can be partially due to the different statistical approaches that are implemented, including trajectory vs. non-trajectory methodologies [18]. Thus, the aims of this systematic review are to: (1) evaluate how non-trajectory analyses inform our knowledge of individual and contextual predictors of later symptoms of anxiety and depression, and (2) evaluate how trajectory analyses inform our knowledge of individual and contextual predictors of change in anxiety and depression symptoms in autistic individuals within longitudinal studies.

## 2. Methods

### 2.1. Search Strategy

A systematic search was carried out by searching two large databases, PsychINFO and PubMed. The search terms were entered in three layers; the first were terms related to autism (i.e., autism, Asperger, ASD, PDD-NOS, “autism spectrum disorder”), the next were related to the longitudinal nature of the relevant articles (i.e., longitudinal, long-term, change, trajectories), and the third was related to mental health and related disorders (i.e., depression, anxiety, “maladaptive behaviors”, “mood disorders”). The exact search strings with MeSH terms and indications of the search location (i.e., abstract/title) can be found in Appendix A. The parentheses in the search indicate different groups of related terms. The only additional limit added during the search was to restrict the search to articles published between 2000 and 2023. The database search took place on February of 2023. This systematic review has been registered with INPLASY (International Platform of Registered Systematic Review and Meta-analysis Protocols) and the registration number is INPLASY2024100068.

**Figure 1:**
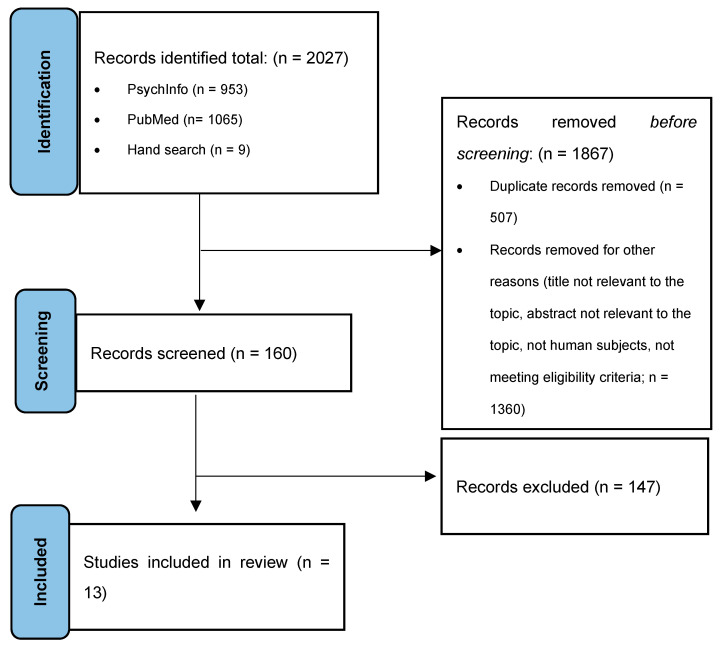
Preferred reporting items for systematic reviews and meta-analysis (PRISMA) flowchart.

The study screening and inclusion process took place in three stages. Articles were first screened for duplicates, then for relevance to the topic and clear violations of the inclusion criteria (i.e., autism-specific, clearly longitudinal, and human subjects) by screening the title and abstract. A final and more rigorous screen was conducted to exclude any additional studies that were missed during the initial screen. During this step, relevant data from each study was extracted to track which studies met all the inclusion criteria, and to indicate why the studies that did not meet the inclusion criteria were excluded. A hand search was then conducted by searching the reference lists of the identified papers to locate additional papers that met inclusion criteria that did not come up in the original search. The search process is shown in the PRISMA flow diagram in Figure 1.

### 2.2. Inclusion Criteria

Studies that met the following inclusion criteria were included in this study:

- Equal to or longer than a three-year follow-up between the first and final timepoint;
- Prospective study;
- Sample size larger than 50 participants;
- Included participants with a confirmed professional diagnosis (use of observational measures, interviews or evidence of a medical diagnosis given by a professional) of ASD, autism, Asperger’s syndrome, or pervasive developmental disorder–not otherwise specified (PDD-NOS);
- Included measures of symptoms of anxiety or depression as the outcome;
- Included behavioral or environmental (e.g., sociodemographic and contextual) predictors.

### 2.3. Exclusion Criteria

Studies that met the following exclusion criteria were excluded from this study:

- Treatment study;
- Only included prenatal or genetic data as predictors;
- Publications not written in English.

Only studies that had a follow-up period of at least three years were included to ensure that we were capturing a sufficiently long interval to see developmental changes. Only studies that included more than 50 participants were included to ensure that the research was appropriately powered to find associations between predictors and the outcomes of interest, given the heterogeneity within autism [19]. Additionally, we excluded studies that only included prenatal data as predictors of outcomes because we were mainly interested in participant behavioral and contextual predictors.

### 2.4. Data Extraction

Data from each article was extracted using a series of questions created for the purposes of this study, including the following: (1) longitudinal sample name and study citation; (2) sample size; (3) mean and standard deviation of age and/or age range; (4) diagnoses were reported for the sample (in addition to autism); (5) mean length of follow-up after the initial timepoint; (6) IQ data, if available; (7) predictors included in the study; (8) construct of interest measured in the study (anxiety and/or depression); (9) outcome measures; (10) trajectory vs. non-trajectory statistical methods; and (11) result summaries.

Many of the studies that were included in the systematic review were secondary data analyses from larger longitudinal samples. As a result, information about the full sample demographics from all the longitudinal cohorts represented in the articles that met eligibility criteria were extracted from the parent studies. Information regarding the original cohort included the following: (1) name of cohort, (2) principal investigators leading the cohort, (3) sample size, (4) diagnoses included, (5) mean age and/or range at entry, (6) average age at the final timepoint, (7) length of study, (8) country of origin, (9) type of sample (e.g., community sample, population-based cohort, etc.), (10) gender ratio, (11) IQ, (12) caregiver education, and (13) participant race.

## 3. Results

### 3.1. Search Results and Study Inclusion

The initial search resulted in the identification of 2027 studies (PsychInfo = 953, PubMed = 1065, hand search = 9). After the first screen, 1867 studies were excluded (duplicates = 507, not meeting inclusion criteria based on title/abstract = 1360). Of the 160 studies that were left, 147 were excluded for the following reasons: treatment study (*n* = 18), not longitudinal (*n* = 20), prenatal study (*n* = 4), genetic study (*n* = 8), insufficient follow-up period (*n* = 29), no confirmed autism diagnosis (*n* = 29), autistic participants not the focus (*n* = 7), and no outcomes of interest (*n* = 28). Additionally, four studies were excluded because they used very general constructs of “emotional problems”, which included symptoms of depression, anxiety, OCD, and additional mental health conditions. While these papers are valuable to the autism and mental health literature, they were excluded in order to focus on symptoms of anxiety and depression as separate outcomes. A total of 13 studies met eligibility criteria and were included in the review. Of the 13 studies, three measured both anxiety and depression using the same participants. The demographic information for all included studies is shown in Table 1.

### 3.2. Characteristics of Longitudinal Cohorts

Seven longitudinal cohorts were represented across the 13 studies that met eligibility criteria. All samples included participants with diagnoses of autism, and some reported on the presence of co-occurring ID and other developmental delays within the sample. Most cohorts began data collection when participants were in early childhood, with only a few beginning in late childhood, adolescence, and adulthood. A wide range of ages are represented in the seven cohorts, and follow-up times within the studies ranged from about 3 to 30 years. The cohorts were primarily based in the United States (*n* = 2), United Kingdom (*n* = 2), and Canada (*n* = 2) but also included samples from Australia (*n* = 1) and Israel (*n* = 1). One of the studies was based in both the United States and Canada and is represented in each country in the previous count (see Table 2). The cohorts include community samples, population-based cohorts, and clinical samples. As expected, each of the cohorts included primarily male participants (from 70 to 90%). A large percentage of caregivers had education levels of a high school diploma or more. Three of the seven cohorts did not report race and/or ethnicity demographics, but the majority of participants in the other studies identified as White. Characteristics of the longitudinal cohorts are shown in Table 2. 

### 3.3. Characteristics of Included Studies 

For the studies included in this review, sample sizes ranged from 65 to 6,091 participants, the larger samples being population-based cohort studies. The 13 studies that met eligibility criteria used data from the following cohorts: four came from the Pathways in ASD study, three from the Early Diagnosis of ASD (EDX) cohort, one from the Special Needs and Autism Project (SNAP) cohort, one from the Avon Longitudinal Study of Parents and Children (ALSPAC), two from a cohort followed by Zachor and Ben-Itzchak, one from the Autism Treatment Network (ATN) Call-Back study, and one from the Australian Child to Adult cohort. Outcomes of anxiety and depression were measured during childhood, adolescence, and adulthood. Multiple outcomes of interest were sometimes included in a single study. Follow-up times within the studies ranged from 4–23 years. Cognitive abilities varied across studies as shown by the wide ranges of IQ scores. Characteristics of the included studies are highlighted in Table 2. A summary of predictors, outcomes of interest, outcome measures, respondents, and results of all studies that met eligibility criteria are described in Table 3 and Table 4.

### 3.4. Anxiety

#### 3.4.1. Results from Studies Using Trajectory Methods

Of the 13 studies, 10 included anxiety as a primary outcome. Four of the ten studies used trajectory analyses, focusing on changes in symptoms of anxiety over time in participants. Of those studies, one showed that symptoms of anxiety decreased from school age to adulthood [25]. Other studies showed variable trajectories with multiple trends of stability, including increases and decreases in anxiety over time within the samples [5,13,22]. For example, one study identified a group with low symptoms of anxiety in childhood that remained low into adulthood, and a group that started with higher levels of anxiety that remained high over time [5].

A number of predictors of trajectories and changes in anxiety were identified across the studies. One study showed that higher levels of executive functioning challenges in childhood were related to increasing levels of anxiety over time into early adulthood [13]. Similarly, a separate study found that children with better adaptive skills at age 9 were less likely to belong in the high anxiety trajectory group from late childhood to adulthood [5]. Other significant predictors of higher anxiety trajectories throughout childhood and from childhood to adulthood included early insistence on sameness [22], caregiver mental health [22], lower parental education [13], and higher IQ scores [5,13]. Being female was associated with higher levels of later childhood anxiety in one sample [22], greater increases in anxiety over time from school age to adulthood but no overall higher levels of anxiety in adulthood compared to males [13], or no associations with any trajectories from childhood to adulthood [5].

#### 3.4.2. Results from Studies Using Non-Trajectory Methods

The remaining six studies used non-trajectory methods, focusing on the association between earlier factors and later symptoms of anxiety, on average, for the entire sample. Earlier psychopathology including high levels of anxiety were reliable predictors of later high levels of anxiety in autistic school age-children [21]. In one large community sample, higher levels of insistence on sameness and RRBs in early childhood predicted later anxiety symptoms in middle childhood [20,21], although in a different sample, they did not predict diagnoses of social or separation anxiety in adolescence [23]. Sleep difficulties in early childhood did not predict later anxiety in school-age children [26].

The association between earlier adaptive functioning and anxiety symptoms found in adolescence in one study was not significant [23]. However, significant associations between higher adaptive functioning in childhood and lower anxiety symptoms in adulthood were found [15], further supporting the findings by Gotham and colleagues [13] and McCauley and colleagues [5]. Findings on the relationship between childhood IQ and later anxiety symptoms were mixed. One study found that higher IQ was associated with higher levels of anxiety symptoms in adolescence [23], while two others found that higher IQ was associated with lower scores of anxiety symptoms in adolescence [28] and adulthood [15]. Of caregiver predictors, earlier symptoms of caregiver mental health were associated with later symptoms of anxiety in adults with autism in one study [15], but no other studies tested this association [20,21,23,26].

### 3.5. Depression

#### 3.5.1. Results from Studies Using Trajectory Methods

Only six studies assessed symptoms of depression as a primary outcome. Half of these studies used non-trajectory analyses. Children with confirmed diagnoses of autism and children without official diagnoses with high autistic traits (determined using measures of social affect and RRBs) had higher depressive symptoms than the general population at age 10, and depressive symptoms continued to increase until age 18 [27]. Additionally, children with autism and high autistic traits who reported being bullied had the highest depressive symptom scores at age 10, which remained elevated throughout adolescence [27]. Autism diagnosis (compared to other developmental delays) showed consistently higher symptoms of depression over time in one study [13]. Similarly, higher social communication autism severity scores (CSS Social Affect score) at age 9 were associated with greater odds of being part of the high depressive symptoms class instead of the low depressive symptom class [5] using the same sample.

Depressive symptoms increased with age for females, similar to anxiety symptoms, but there were no significant gender differences in symptoms of depression between males and females in adulthood [5,13]. The associations between IQ and depressive symptoms were unclear; higher IQ at age 5 was found to be associated with higher odds of belonging to the high depression group in adulthood in one study [5], while no significant associations were found in another that used the same longitudinal cohort but slightly different sample and different longitudinal methods [13]. One study found that lower caregiver education predicted increasing depressive symptoms in adults, but only for those with IQ scores of less than 70 [13]. More severe ADHD and anxiety symptoms at age 9 were positively associated with the high depressive symptoms class compared to the low depressive symptoms class [5]. In one study, more severe executive functioning difficulties (EF) were associated with higher symptoms of depression during school age, but with slower-growing depressive symptoms over time in this group compared to those with less severely affected EF skills [13]. Similarly, worse adaptive skills at age 9 predicted an increased risk of belonging to the class that had high depressive symptoms over time from childhood to adulthood [5].

#### 3.5.2. Results from Studies Using Non-Trajectory Methods

Similar to findings from the trajectory analyses by Gotham and colleagues [13] and McCauley and colleagues [5], in non-trajectory analyses, higher adaptive skills predicted lower symptoms of depression in adulthood [15]. In a separate study by Forbes and colleagues [24] using the same sample, depressive symptoms in adulthood were not related to autism severity, adaptive skills, IQ, irritability, or hyperactivity in childhood and adolescence, including teacher reports of behavioral and emotional problems at ages 14 or 17. In another sample, higher irritability and lower peer relationship scores in preschool age were associated with higher symptoms of depression in adolescence [14]. Finally, of the sociodemographic and contextual factors, maternal mental health and neighborhood deprivation were significant predictors of later symptoms of depression in autistic adolescents and adults. Higher maternal symptoms of depression measured when children were three were a significant predictor of higher symptoms of depression in autistic adolescents [14]. Furthermore, worse maternal mental health measured in childhood predicted higher symptoms of depression in adults [15]. One study found that greater neighborhood deprivation in childhood was related to lower depressive symptoms in adulthood, but the association was weak [15].

## 4. Discussion

Higher rates of co-occurring psychological conditions in autistic people compared to the general population have been well-established in the literature [4,31]. Furthermore, these co-occurring psychological conditions are linked to individuals’ quality of life and well-being [5,8,13]. We undertook a systematic review to evaluate the unique contributions of trajectory and non-trajectory analyses to our understanding of symptoms of anxiety and depression in autistic individuals across their lifespan and to identify unique predictors of those outcomes. These aims are critical for identifying developmental periods where increased support and targeted interventions may be beneficial.

### 4.1. Trajectory Analyses in Autism Research

The analytic methods used were quite variable across the included studies, even within trajectory and non-trajectory models. Some studies used growth curve modeling, group-based trajectory modeling and structural equation modeling followed by regression analyses, while other studies analyzed their data using simple correlations between entry and exit variables. There is support for the statistical equivalence of the various approaches [32]. However, different methods used to model data can influence the interpretation of the relationships between variables and our ability to see patterns of change in behaviors over time [33]. A strength of trajectory models is that they allow us to understand whether overall levels and changes in anxiety and depressive symptoms are homogeneous across time in the population of interest or whether there is variability in the development of symptoms. Based on the trajectory studies included in this review, it is clear that there are a range of different trajectories (identified as multiple groups with varying levels of anxiety and depression) that change differently over time in autistic populations. Despite this, trajectory-based analytical methods are used less often than expected in longitudinal autism research, likely due to the large sample sizes that are required to secure enough statistical power [18].

### 4.2. Predictors of Mental Health Outcomes

One clear pattern that emerged from the included studies was that early mental health challenges are strong predictors of trajectories of later mental health challenges. Earlier symptoms of anxiety or depression are consistently associated with higher symptoms of anxiety and depression later in life. There was some inconsistency in the relationship between adaptive skills and later symptoms of anxiety in adolescence [23], but overall, worse adaptive skills predict worse symptoms of anxiety and depression trajectories and outcomes in adulthood [5,13,15]. Targeting adaptive skills early in development could be a way to support mental health outcomes in adulthood. There are few interventions specifically targeted at improving adaptive skills in children with autism; most interventions are focused on increasing adaptive skills in adulthood [34]. It is possible that there is a window to support both adaptive skills and symptoms of anxiety and depression in late school age or early adolescence before mental health challenges become clinically significant. Learning more about the effects of intervention on adaptive skills in childhood and the mediating effects of improving adaptive skills on later mental health outcomes is an area of research that requires further exploration.

IQ has consistently been identified as a strong predictor of many more positive adult outcomes on average [35]. The varying results across studies using both trajectory and non-trajectory methods suggest that the relationships between IQ and symptoms of anxiety and between IQ and symptoms of depression remain less clear. The inconsistent relationships between IQ and mental health challenges could be due to several reasons, including but not limited to differences in respondents (self vs caregiver report) and/or low rates of inclusion of individuals who are less cognitively able in longitudinal samples.

The relationship between gender and mental health symptoms needs further exploration as well. Variability in rates of anxiety and depressive symptoms across males and females in the literature could be attributed to ascertainment differences, including oversampling of males in general and oversampling of females with lower IQs in face-to-face research studies [36,37]. Furthermore, females identified in adulthood are clinically different on average compared to those that are identified in childhood (e.g., higher IQ and levels of anxiety) [38]. While it is costly and time intensive, matched samples for gender, IQ, and age are needed to systematically assess whether there are gender differences in mental health symptoms and additional clinical factors that influence these relationships (e.g., age, IQ, adaptive skills, etc.).

Autistic individuals have higher symptoms of depression over time than the general population, while insistence on sameness and RRBs (as opposed to an autism diagnosis) in childhood predicts later anxiety symptoms. Additionally, having positive peer relationships during school age seems to protect against symptoms of depression later in life in autistic adolescents [14,16,27]. Studies should assess whether social skill interventions that target social skills in children and adolescents [39] indirectly influence symptoms of depression later in life through the mediating role of social relationships. An additional predictor of depressive symptoms in autistic adolescents and adults was earlier maternal mental health. This highlights the potential bidirectional relationship between parent and child mental health outcomes and the need to support family units.

### 4.3. Limitations

The results presented here should be considered with a few limitations. The included studies covered a wide range of development (i.e., childhood, adolescence, and adulthood) and specific periods of development varied across studies. It may be more appropriate to compare studies within a particular age range. This is difficult to do, however, given the relatively few longitudinal studies available. Additionally, the studies used different measures for their predictors and outcomes, and varied in reporters (e.g., teacher, parent or self-report). For example, one study by Ben-Itzchak and colleagues [23] used SCARED to identify symptoms of separation anxiety and social anxiety, while the majority of studies relied primarily on the anxiety subscale of the CBCL and ABCL. Moreover, there is still little evidence that different measures and different reporters of these outcomes tap into the same constructs. Future work is needed to link and validate the use of common measures of anxiety and depression for autistic people [40,41]. Additionally, the majority of the included literature used questionnaire data to measure changes in symptoms of anxiety and depression, as opposed to using professionally provided diagnoses.

## 5. Conclusions

This is the first review to synthesize data on early predictors of later symptoms of anxiety and depression in autism using longitudinal data. There are many factors, both individual and contextual, that have been assessed as predictors of mental health outcomes, both cross-sectionally and longitudinally, in this population. This review provides a new perspective on the consistency, or lack thereof, of early predictors of symptoms of anxiety and depression. Some factors reliably predict later symptoms of anxiety and depression such as childhood adaptive skills and peer relationships, and others are less consistent, like IQ. Additionally, this review provides solid evidence that there are different trajectories and different patterns of mental health symptoms over the lifespan, supporting the importance of acknowledging that autism is truly a spectrum, as well as a developmental condition that changes over time in different ways for different people. Because of the heterogeneity in autism across almost all behaviors and characteristics that we measure in clinical practice and research (e.g., age, gender, IQ, adaptive skills, etc.), large and diverse longitudinal samples are necessary to appropriately capture different profiles and patterns of development across the lifespan. Understanding these outcomes is the first step to being able to implement interventions at the right time to offset the effects on anxiety and depression outcomes.

## Figures and Tables

**Table 1:** Demographic information of included articles.

First Author and Year	Longitudinal Sample	n	Mean Age	Diagnoses	Approximate Length of Follow-Up	IQ
[20]	Pathways in ASD	421	Study intake = 3.4 years, SD = 0.8, T1 = 4.0 years, SD = 0.8, T2 = 6.6 years, SD = 0.3, T3 = 8.7 years, SD = 0.20, and T4 = 10.8 years, SD = 0.24	Autism	7.4 years	Mean IQ = 59.08 (SD = 24.97), range = [10.00–154.00]
[21]	Pathways in ASD	421	T1 = 3.3 years, T8 = 10.7 years	Autism	7.4 years	Low-stable insistence on sameness group—mean = 59.2 (SD = 24.1), moderate-increasing insistence on sameness group—mean = 58.2 (SD = 24.7), high-peaking—mean = 66.7 (SD = 33.8)
[22]	Pathways in ASD	346	T1 = 40.1 months (SD = 8.9)	Autism	6 years	Median IQ = 58.00 (range = 43.0–72.0)
[23]	Zachor and Ben-Itzchak	65	T1 = 25.6 months (SD = 4.5), T2 = 164.8 months (SD = 22.7)	Autism	11.7 years	T1 range, 49–127, T1 mean = 73.71 (SD = 18.75); T2 range, 40–146, T2 mean = 70.02 (SD = 24.98)
[14]	Pathways in ASD	390	T1 = 3.41 years (SD = 0.76), T2 = 3.99 years (SD = 0.79), T3 = 4.51 (SD = 0.76), T8 = 10.76 (SD = 0.24), adolescence (T9) = 13.84 years (SD = 0.83)	Autism	10 years	T1 standard scores = 57.51 (SD = 26.10)
[15]	Special Needs and Autism Project (SNAP)	121	median age = 11.6 years (range = 10.9–12.3) at entry, median age = 23.0 years (range = 22.5–23.7) in adulthood	Childhood autism, Asperger syndrome, pervasive developmental disorder not otherwise specified, or atypical autism	11 years	T1 mean IQ = 73.2 (SD = 24.4)
[24]	EDX	123	Median age = 2.6, range = 1.3–11.8	Autism and other nonspectrum developmental delays	23 years	VIQ median = 37 (range = 10–128), NVIQ median = 75 (range = 13–132)
[13]	EDX	165	T1 10.7 years (SD = 2.5), age range for ASD = 5.9–19.8 years, mean age for NSDD = 12.3 years (SD = 2.3), range = 7.6–16.7	Autism, and nonspectrum developmental delays (i.e., intellectual disability, language delay, or issues such as severe attention-deficit hyperactivity disorder [ADHD])	7.65 years	ASD range = 10–141, mean = 56.3 (SD = 40.1); non-ASD delays range = 14 – 139, mean = 79.6 (SD = 33.5)
[25]	Australian Child to Adult cohort	T1: 119, T5: 89	Time 1 = 8.7 years (SD = 4.3 years), time 5 = 24.8 years (SD = 4.7 years)	Autistic disorder	16 years	N/% of participants with average IQ = 11 (9.2%), borderline = 16 (13.4%), mild intellectual disability = 29 (24.4%), moderate intellectual disability = 46 (38.7%), severe ID = 17 (14.3%)
[26]	The Autism Treatment Network (ATN) Call-Back study	437	Overall mean age = 5.07 years, (SD = 2.14 years), age range = 2–10 years at entry; Group ages 2–3 mean = 2.98 years (SD = 0.58 years); Group ages 4–10 mean = 6.35 years (SD = 1.70 years)	Autism	3.8 years	Overall sample mean = 76.24, (SD = 22.23), range = 40 –132; Group ages 2–3 mean = 66.76 (SD = 17.59); Group ages 4–10 mean = 81.64 (SD = 22.82)
[5]	EDX	194	T1—late childhood (M = 9.36 years, range 5.67–11.83), T2—early adolescence (M = 14.93 years, range 13.42–15.92), T3—late adolescence (M = 17.40 years, range 16.00–18.67), T4—early adulthood (M = 20.25 years, range 19.00–22.75), T5: adulthood (M = 25.73 years, range 23.17–30.08)	Autism and other developmental delays	23 years	Low decreasing ADHD symptoms—age 9 mean VIQ = 69.26 (SD = 38.23), High decreasing ADHD symptoms—age 9 mean VIQ = 46.9 (SD = 34.85), Low stable anxiety symptoms:—age 9 mean VIQ = 48.77 (SD = 34), High stable anxiety symptoms—age 9 mean VIQ = 90.98 (SD = 32.38), Low stable depressive symptoms—age 9 mean VIQ = 61.71 (SD = 38.31) High/fluctuating depressive symptoms—age 9 mean VIQ = 57.4 (SD = 38.79)
[27]	The Avon Longitudinal Study of Parents and Children (ALSPAC)	6091 and 3168 (different analyses)	T1 = 10 and last timepoint = 18	Autism, Asperger’s, and typically developing	8 years	NA
[28]	Zachor and Ben-Itzchak	69	T1 = 2.2 years (SD = 0.5), T2 = 13.10 years (SD = 1.11)	Autism	11.06 years	Overall group mean = 70.80 (SD = 24.89), Low-functioning ASD group mean = 54.56 (SD = 13.99), High-functioning ASD group mean = 96.07 (SD = 8.88), Best outcome group mean = 99.0 (SD = 17.79)

Note: ASD—autism spectrum disorder, IQ—intellectual quotient, VIQ—verbal intellectual quotient, NVIQ—nonverbal intellectual quotient, ID—intellectual disability, NA—not available, SD—standard deviation, T[1–5]—time [1–5], ADHD—attention-deficit hyperactivity disorder, and CSS—calibrated severity scores.

**Table 2:** Characteristics of the longitudinal cohorts.

Cohort Name	Primary Investigators	Sample Size	Diagnoses	Average Age at Entry	Average Age at Final Timepoint	Length of Study	Country	Type of Sample	Gender Ratio	IQ	Caregiver Edu	Race
Pathways in ASD	Peter Szatmari	424	Autism	3 years	12–19 years (collection ongoing)	18 years	Canada	Inception cohort/community sample	83.7% male	M-P-R was 59.1 (SD = 27.3)	Professional certificate or above, 65.7%	73.5% White, 26.5% Other
Early Diagnosis of ASD (EDX) study	Catherine Lord	253 (consecutive referrals)	Autism and other developmental delays	2 years	34 years	32 years	United States	Community/clinic referred sample	86% male	62% VIQ ≤ 70, range 3–141	46% college degree or above	72% White, 26% Black, 2% Other
Special Needs and Autism Project (SNAP) cohort	Emily Simonoff, Tony Charman, Andrew Pickles	158	Autism	12 years	23 years	11 years	United Kingdom	Population-based cohort	3.3:1 (male to female)	55% IQ < 70, mean = 69.4 (SD = 24.1)	61% >high school diploma	NA
The Avon Longitudinal Study of Parents and Children (ALSPAC)	University of Bristol (Demo taken from Culpin and colleagues	166 (diagnosed with ASD by 11 years)	Autism	0 years (birth)	30 years	30 years	United Kingdom	Population-based cohort	80% male	96.4% IQ >70	54% O-level or less (high school or less)	NA
Zachor and Ben-Itzchak	Zachor and Ben-Itzchak	66	Autism	2.13 years	13.8 years	11.67 years	Israel	Clinical sample	93% male	Mean IQ = 73.71 (SD = 18.75), range = 49–127	11–22 years of maternal education (M = 15 years, SD = 2.6)	100% White
The Autism Treatment Network (ATN) Call-Back study	Autism Speaks	437	Autism	5.07 years	8.90 years	3.8 years	United States and Canada	Community sample	82.6% male	Mean IQ = 76.24 (SD = 22.2), range = 40 to 132	29.3% caregivers attended some college and 31.1% attained a bachelor’s degree	86% White
Australian Child to Adult cohort	Gray and colleagues	119	Autism	8.7 (range, 2.8–19.8)	24.8years	>18 years	Australia	Community sample	82.4% male	77.4% with ID	NA	NA

Note: ASD—autism spectrum disorder, IQ—intellectual quotient, VIQ—verbal intellectual quotient, NVIQ—nonverbal intellectual quotient, ID—intellectual disability, NA—not available, SD—standard deviation, T[1–5]—time [1–5], ADHD—attention-deficit hyperactivity disorder, and CSS—calibrated severity scores.

**Table 3:** Predictors and results of included articles on anxiety.

First Author and Year	Significant Predictors	Stats Used	Outcome Measures	Selected Results
[20]	Insistence on sameness	Non-trajectory; cross-lagged panel models (CLPMs) built via structural equation modeling	Anxiety Problems subscale from the CBCL and the Repetitive Behavior Scale-Revised, Ritualistic/Sameness subscale	Insistence on sameness predicted future anxiety from T2 to T3 (7–9 years) and from T3 to T4 (9–11 years), even when controlling for concurrent anxiety levels, baseline anxiety, and baseline insistence on sameness. After controlling for language, IQ, age at diagnosis, and sex, the results remained the same.
[22]	Higher baseline insistence on sameness, parenting stress and being female	Trajectory; quadratic growth curves	Anxiety Problems subscale from the CBCL and Autism Diagnostic Interview-Revised (ADI-R) insistence on Sameness subscale	Four groups emerged from the anxiety data: a low-increasing group (51.0% participants), a moderate-decreasing group (16.2% participants), a moderate-increasing group (19.6% participants) and a high-stable group (13.1% participants). Higher baseline insistence on sameness, parenting stress and being female significantly predicted being part of higher-level anxiety trajectories.
[21]	Repetitive behaviors in early childhood and earlier anxiety symptoms	Non-trajectory; logistic regression analyses	Anxiety Problems subscale from the CBCL	Moderate and severe repetitive behavior at ages 2–5 years significantly predicted elevated anxiety symptoms at ages 8–11 after adjusting for confounders. Baseline anxiety symptoms on the CBCL significantly predicted middle childhood anxiety (for each 1-point increase on this measure, the odds of later anxiety symptoms increased by 1.2)
[23]	Younger age, cognitive ability	Non-trajectory; hierarchical regression analyses	Screen for Child Anxiety Related Emotional Disorders (SCARED)	Younger age was associated with increased separation anxiety scores at T2 (about age 14). Cognitive ability at T1 contributed 11.0% of the explained variance. Lower cognitive ability at time 1 (about age 2) was associated with the greater separation anxiety scores at time 2. Adaptive skills, anxiety in the family, and RRBs at time 1 were non-significant predictors of separation anxiety at time 2. The social anxiety model was not statistically significant.
[15]	Childhood IQ, VABS daily living skills, VABS socialization, better maternal mental health, and worse VABS communication	Non-trajectory; negative binomial regression, linear regression	The following subscales of the Strengths and Difficulties Questionnaire (SDQ): conduct problems, emotional problems, and hyperactivity subscales	Here, 9% of young adults were identified as moderately anxious, and 1% as severely anxious. Significant predictors of lower anxiety in adulthood were higher childhood IQ, VABS daily living skills, VABS socialization, better maternal mental health, and worse VABS communication. Prediction of later anxiety from childhood predictors was poor (r-squared close to zero or negative).
[13]	Executive function, gender, autism diagnosis, age, higher symptoms of anxiety, and maternal education (for less verbal adults)	Trajectory; generalized mixed-effects models	The Affective subscale from the CBCL and the Depressive subscale from the parent-rated Adult Behavior Checklists (ABCL) Communication and Anxiety Disturbance (DBC-Anxiety) and Depressive (DBC-Depressive) subtotals of the Developmental Behavior Checklist-Adult for participants with less than fluent speech	Higher VIQ and having a diagnosis of autism were associated with a higher linear increase of anxiety. For anxiety, males started with higher symptoms at study onset compared to females, but females had greater increases in symptoms over time. This resulted in no differences in anxiety symptoms at age 21. A significant childhood predictor of anxiety slopes included executive function at age 9. For a smaller subset of less verbal participants (N = 44): There was no significant change over time in anxiety symptoms, and no effects of VIQ or gender. A significant interaction between age and maternal education arose; mothers with lower levels of education reported greater symptoms of anxiety over time.
[25]	Earlier behavioral and emotional problems	Trajectory; regression analysis trajectory	The parent (DBC-P) and Adult (DBC-A) versions of the Developmental Behavior Checklist	Of the five subscales of the DBC (Disruptive, Self-Absorbed, Anxiety, Communication Disturbance, and Social Relating Problems), all except self-absorption showed a significant decline in scores over time.
[26]	None	Non-trajectory; linear regression and correlation	The Child Behavior Checklist (CBCL) [29], Anxiety Problems DSM-Oriented Scale	Sleep and anxiety were significantly correlated at entry for both age groups (2–5 years and 4–10 years), but there were no significant longitudinal associations between sleep problems and anxiety. Sleep at T1 (2–5 years and 4–10 years) did not predict anxiety at T2 (6–7 years and 8–14 years), controlling for gender, age at baseline, IQ, caregiver education, and time between assessments.
[5]	Higher VIQ and higher adaptive skills in childhood	Trajectory; group-based trajectory modeling, multinomial logistic regression models, ordinal regressions	CBCL, the ABCL, Social and Emotional Functioning Interview—Revised (SEFI-R)	Anxiety outcomes data resulted in two trajectory groups: (1) A Stable Low Anxiety Symptoms group with low levels of anxiety overall through adulthood (25 years) (74.23% of sample); (2) A Stable High Anxiety Symptoms group that maintained high levels of anxiety over time (25.77% of sample). The two classes differed by gender; 25% females were in the low anxiety group and only 10% in the high stable group. The gender differences were not significant after accounting for VIQ. Higher VIQ at 5 and 9 years predicted membership in the Stable High Anxiety Symptoms class. Higher levels of adaptive skills at age 9 were associated with decreased odds of being in the Stable High Anxiety Symptoms class. Neither depressive symptoms nor ADHD symptoms at baseline were predictive of the Stable High Anxiety Symptoms trajectory probability.
[28]	Cognitive abilities greater than or equal to 80, and autism diagnosis	Non-trajectory; ANOVA	Screen for child anxiety related emotional disorders and the Conners, Third Edition (Conners 3)	A significantly higher percentage of participants in the high functioning group reached the clinical cut-off score in at least one SCARED subdomain (69%) compared to the best outcome group (27.3%). Moreover, 57.1% of the lower functioning autism group reached the clinical cut-off score in at least one SCARED subdomain but was not significantly different from the other two groups. The best outcome group differed significantly in the Conners-3 socialization subdomain and had less severe difficulties compared to the other two groups, but there were no differences in the panic, generalized, separation, social, and avoidance of school SCARED subdomains.

Note. ASD—autism spectrum disorder, IQ—intellectual quotient, VIQ—verbal intellectual quotient, NVIQ—nonverbal intellectual quotient, ID—intellectual disability, NA—not available, SD—standard deviation, T[1–5]—time [1–5], ADHD—attention-deficit hyperactivity disorder, and CSS—calibrated severity scores.

**Table 4:** Predictors and results of included articles on depression.

First Author and Year	Significant Predictors	Stats Used	Outcome Measures	Selected Results
[14]	Earlier irritability and fewer peer relationships	Non-trajectory; structural equation models and mediation models	Depressive Problems subscale of CBCL	Early irritability significantly predicted depressive symptoms at age 14, controlling for baseline depressive symptoms. No other baseline covariates predicted depressive symptoms at 14 years. Early childhood irritability significantly predicted lower peer relationship scores at age 10. Lower peer relationship scores were associated with higher depressive symptoms at age 14. There was a significant indirect association of peer relationships. Peer relationships mediated 13% of the observed association between irritability and depression. Educational engagement was not a significant predictor of depression. When ADHD symptoms were included as a covariate, early childhood irritability and maternal depression significantly predicted later youth depression. In the mediation model, there was no significant direct effect of ADHD on depression or indirect effects. The direct effect between peer relationships and later depression remained significant.
[15]	Daily living skills, better maternal mental health, and greater neighborhood deprivation	Non-trajectory; negative binomial regression and linear regression	The following subscales of the Strengths and Difficulties Questionnaire (SDQ): conduct problems, depression, emotional problems, and hyperactivity subscales	A total of 11% of young adults were identified as moderately depressed, and 1% severely depressed. Significant predictors of lower depression in adulthood included daily living skills, better maternal mental health, and greater neighborhood deprivation. Prediction of later depression from childhood predictors was poor (r-squared close to zero or negative).
[24]	No significant predictors	Non-trajectory; odds logistic regression and linear regression	Adult measures: The Beck Depression Inventory-II (BDI- II)	There was little success in predicting depression from the predictors at any age even after including teacher reports of behavioral and emotional problems at ages 14 or 17.
[13]	Age, autism diagnosis, gender, executive function at age 9, and maternal education (for less verbal participants)	Trajectory; generalized mixed-effects models	The Affective subscale from the CBCL and the Depressive subscale from the parent-rated Adult Behavior Checklists (ABCL), Communication and Anxiety Disturbance (DBC-Anxiety) and Depressive (DBC-Depressive) subtotals of the Developmental Behavior Checklist-Adult for participants with less than fluent speech	Age and having a diagnosis of autism were associated with higher linear growth of depression. For depression, males started with higher symptoms at study onset compared to females, but females had greater increases in symptoms over time. This resulted in no differences in depressive symptoms at age 21. There were no effects of VIQ on depression. Significant childhood predictors of depression slopes included executive function at age 9. For a smaller subset of less verbal participants (N = 44), the following were found: For depression, there was a main effect of age, meaning that symptoms significantly increased at age 18. There was a significant interaction between age and gender. At the onset of the study, depressive symptoms were no different between males and females, but significantly increased for females over time. Additionally, there was a significant interaction between age and maternal education; participants of mothers with lower levels of education reported greater symptoms of depression over time.
[5]	Verbal intellectual quotient at age 5, autism severity scores at age 9, adaptive skills, and baseline levels of ADHD symptoms and anxiety	Trajectory; group-based trajectory modeling, multinomial logistic regression models, and ordinal regressions	CBCL [29], the ABCL [30], Social and Emotional Functioning Interview—Revised (SEFI-R)	Depression Outcomes Data resulted in two trajectory groups: (1) A Stable Low Depressive Symptoms group with low levels of depressive symptoms overall through adulthood (68.04% of sample); (2) A High/Fluctuating Depressive Symptoms group that had stable levels of depression from T1 to T2, increased from T2 to T4, and decreased from T4 to T5 (31.9%). The two groups did not differ by race, gender, ASD diagnosis, or caregiver education. Higher VIQ at age 5 and higher CSS scores at age 9 were independently associated with higher odds of being in the High/Fluctuating Depressive Symptom class. Higher adaptive skills at age 9 were negatively associated with the odds of being in the High/Fluctuating group. Baseline levels of ADHD symptoms and anxiety symptoms were positively associated with membership in the High/Fluctuating Depressive Symptoms class over the Stable Low Depressive Symptoms class.
[27]	Reports of bullying at ages 8, 10, and 13 (latent factor)	Trajectory: mixed-effects linear growth models	The Short Mood and Feelings Questionnaire (SMFQ); The Clinical Interview Schedule–Revised (CIS-R) at age 18	Children with autism and autistic traits had higher depressive symptom scores than the general population at age 10, increasing until age 18. No evidence of a significant association between autism and a depression diagnosis at age 18 was observed. Children with autism and autistic traits who also reported being bullied had the highest depressive symptom scores at age 10, which remained elevated throughout adolescence.

Note: ASD—autism spectrum disorder, IQ—intellectual quotient, VIQ—verbal intellectual quotient, NVIQ—nonverbal intellectual quotient, ID—intellectual disability, NA—not available, SD—standard deviation, T[1–5]—Time [1–5], ADHD—attention-deficit hyperactivity disorder, and CSS—calibrated severity scores.

## Data Availability

Search Criteria provided in Appendix A.

## Appendix A

Search terms
PubMed: (((“autis*”[Title/Abstract] OR “asperger*”[Title/Abstract] OR
“asd”[Title/Abstract] OR “PDD-NOS” [Title/Abstract] OR “autism spectrum disorder”[MeSH
Terms])))
AND
(((“longitudinal”[Title/Abstract] OR “long-term”[Title/Abstract] OR
“change”[Title/Abstract] OR “trajector*”[Title/Abstract])))
AND
(((“depress*”[Title/Abstract] OR “anxiety”[Title/Abstract] OR “mood disorders”[MeSH
Terms] OR “Anxiety”[MeSH Terms] OR “Depression”[MeSH Terms] OR “maladaptive
behaviors”[Title/Abstract])))
Psych info: (tiab(autis*) OR tiab(asperger*) OR tiab(asd) OR tiab(PDD-NOS) OR
MAINSUBJECT.EXACT.EXPLODE(“Autism Spectrum Disorders”))
AND
(tiab(longitudinal) OR tiab(long-term) OR tiab(change) OR tiab(trajector*))
AND
(tiab(anxiety) OR tiab(depress*) OR tiab(“maladaptive behaviors”) OR
(MAINSUBJECT.EXACT(“Anxiety”) OR MAINSUBJECT.EXACT.EXPLODE(“Anxiety”)) OR (MAINSUBJECT.EXACT.EXPLODE(“Affective Disorders”) OR
MAINSUBJECT.EXACT(“Affective Disorders”)) OR
(MAINSUBJECT.EXACT.EXPLODE(“Depression (Emotion)”) OR
MAINSUBJECT.EXACT(“Depression (Emotion)”)))
